# Torturing environments and multiple injuries in Mexican migration detention

**DOI:** 10.1057/s41599-022-01252-y

**Published:** 2022-08-08

**Authors:** Julia Manek, Andrea Galán-Santamarina, Pau Pérez-Sales

**Affiliations:** 1 Goethe-University Frankfurt/Main, Frankfurt, Germany; 2 Sir[a] Centre, Madrid, Spain

**Keywords:** Psychology, Geography, Sociology

## Abstract

Mexico’s role in the US-Central American migration regime is threefold: not only is it a country of origin, and a transit country, but also increasingly becoming a receiving country for migrants who flee from violence, insecurity and poverty. The Mexican state responds with detention enforcement. Clinical research usually puts emphasise on the negative impact of detention enforcement on the detainees‘ mental health. Yet, it often disregards the spatial configurations of detention centres and their socio-political context. This study aims to fill this gap by analysing how such factors create harmful environments that affect both the detainees‘ mental health and their social life in Mexico’s migration detention centres. The study’s mixed method approach builds on semi-structured interviews with a sample of *N* = 56 migrants of diverse nationalities and varying socioeconomic status of whom 22 were still detained while 34 had been released. The interviews include the Torturing Environment Scale (TES), a novel instrument for the analysis of detention environments, as well as clinical psychological measures of emotional distress. Additional *n* = 10 in-depth interviews with human rights advocates to explore the interconnections between the detention environments, their impact on mental health, and Mexican migration politics. Facultative counter-mappings of the detention centres complement the interviews. Without exception, all interviews of detainees underline that the manipulation of detention conditions creates torturing environments that cause harm to basic physiological and psychological needs. A comparison between detained vs. released interviewees revealed lasting feelings of fear and shame. The study emphasises that immigration detention immobilises migrants in a necropolitical limbo, which destroys hope as much as human integrity. It indicates that detention is part of deterrence politics, which perpetuates harm and inequality through detention and deportation. Highlighting structural human rights violations, the findings stress the need to review current migration policies.

## Introduction

Detention of undocumented migrants and asylum seekers has become a globalised norm of the ruling. Along the borders of both arrival and transit states, detention camps emerge. Independently of its geographical place, there is various evidence that migration detention has a severe impact on the detainees’ mental health outcomes (e.g. Hallas et al., 2007; Robjant et al., 2009; Steel et al., 2006; van Hout et al., 2020). What migration researchers call politics of deterrence, clinical studies call post-migration stressors (Hynie, 2018).

Thus, migration detention is a primary research field in which the perspectives of migration scholars and clinical psychologists meet. A linking concept evolves in the study of torture as a means of deterrence (e.g. Bhatia and Bruce-Jones, 2021; Brooker et al., 2017; Pérez-Sales, 2018). Still, research often remains within its disciplinary boundaries. The present study aims at crossing these boundaries with an interdisciplinary investigation of migration detention as torturing environments in the particular case of the US-Mexican migration regime^1^. In this study, we geographically focus on the Mexican territory, especially on its Southern border. While the mental health impact of the detention facilities on the US side of the border has been scrutinised from multiple disciplines (e.g. MacLean et al., 2019; Ochoa et al., 2010; Saadi et al., 2020), clinical research on the Mexican immigration detention facilities is scarce.

This research explores the environment of the so-called “*estaciones migratorias”*^2^. It investigates the creation of harmful conditions and how migration detention affects a detainee’s mental health and social life. To answer these ranging questions, this study uses a mixed method approach. Based on a sample of *N* = 56 immigration detainees of diverse nationalities and varying socio-economic statuses, the research applies the Torturing Environment Scale (TES, Pérez-Sales, 2016), as a special instrument to analyse detention environments and the emotional impact on detainees. Clinical psychological measures of mental health were adapted to the particular context (Grupo Impulsor Contra la Detención Migratoria y la Tortura, 2020). To further explore the interconnections between the configurations of the migration detention centres and their impacts and their role within the broader migration regime, additional *n* = 10 in-depth interviews were conducted with an expert group of human rights actors. Facultative psycho-geographical mappings (Campos-Delgado, 2018; Gieseking, 2013; Manek and Fernández de la Reguera, 2022) of the respective *estación migratoria* accompanied the interviews that visualised the spatial configuration of a possible torturing environment.

Our findings emphasise that detention in the *estaciones migratorias* meets the criteria of a torturing environment (Pérez-Sales et al., 2021) with lasting impacts for the mental health of the detainees. The mixed method results suggest that they reach beyond the duration of detention and lead to prolonged mental distress, social exclusion and inequality.

## Context

Mexico’s role in the US-Central American migration regime is threefold: not only is it a country of origin and a transit country, but it is also increasingly becoming a receiving country for migrants. Migration from the Central American states to the US has been conceptualised rather exclusively in the context of labour migration at the beginning of the century. However, this predominant narrative has changed: It is now understood as a form of flight from precarious economic conditions, insecurity, extortion and violence (Varela, 2018) to which the Mexican state responds with detention and deterrence (Colectivo de Monitoreo de Derechos Humanos en el Sureste Mexicano, 2019; Gutiérrez López et al., 2019). Lately, Campos-Delgado (2021) has framed these conditions and treatments in the Mexican detention centres as clear politics of deterrence and forms of punishment—although the *estaciones migratorias* are not supposed to be a punitive institution^3^.

### A vertical border framework

As a central transit region towards North America, especially for migration to the US, Mexico has increasingly become its externalised “vertical” border (Bahena Juarez, 2019; Basok and Candiz, 2020; Bermúdez, 2019; Garibo, 2016). Varela (2017) emphasises that Mexico is “the world’s most crossed, monitored and militarised border”. As a result, Mexico has become one of the countries with the highest rates of detention and deportation worldwide (Global Detention Project, 2021). Built at strategic points, the *estaciones migratorias* have become nodes on the “veins of migration”, lining the migratory routes along which migrants move through Mexico and criss-cross the entire country (Vogt, 2017). Especially the South-Eastern border region of the Mexican territory became a focal zone of migration control: More than half of the nearly 60 *estaciones migratorias* are situated along the 970 km part of the southern frontier that Mexico shares with Guatemala (Cornelio Landero, 2015; León Ang and Lacruz,^ 2021^).

Legally, this scenario is framed by the Mexican migration law: it describes the detention system in the *estaciones migratorias* and their administration by the Mexican migration authority *Instituto de la Migración* (INM) as a form of “accommodation” (Cámara de Diputados del H. Congreso de la Unión, 2014). It must comply with human rights standards, including access to medical and psychological health facilities, prevention of overcrowding conditions and respect for the human rights of the persons admitted, such as access to information or the guarantee of the presence of a translator. However, a terminological comparison indicates that the inclusive discourse used by Mexican legislators still contains a security paradigm: it uses the language of protection for the interests and rights of Mexican citizens and as such, criminalises migrants who are labelled as irregular (López, 2018).

### Massive migration and detention in Mexico

A turning point for the detention practices of the Mexican migration politics was marked in 2018, when thousands of people, mainly Central Americans, formed a veritable exodus and overran the southern border before heading across Mexico and towards the US. Their physical movement had been ended forcefully at the Northern borders of Mexico by armed military forces. Subsequently, a humanitarian crisis emerged on the Mexican side of the border. After the first massive movements of caravans, border practices changed substantially (Colectivo de Monitoreo de Derechos Humanos en el Sureste Mexicano, 2019). Securitisation, militarisation and criminalisation were augmented simultaneously (Gutiérrez et al., 2019); troops of the newly created national guard were not only sent to the borders of Mexico’s South and North—but also sent to reinforce the control and management of the *estaciones migratorias* by the civil-like *INM*. From the beginning of 2019 up to the second half of the same year, the detention rate doubled (Cattan, 2019) and the deportation rate tripled (Cullell, 2019).

In the last decade, detention numbers rose steadily. They reached a peak with the arrival of the migrant caravans, but decreased with the outbreak of the pandemic. In 2019, the Global Detention Project (2021) recorded 182,940 detentions. In 2020, in the first year of the Covid-19 pandemic, 59,155 detentions were documented. The detainees are mainly citizens from Central American states—Honduras, El Salvador, Guatemala and Nicaragua—but people from India and West Africa are amongst the detained as well (Global Detention Project, 2021). The detention population consists mainly of cisgender men, but also includes cisgender women, queer persons, adolescents and children (Gárcia, 2022; Léon Ang and Lacruz,^ 2021^).

Although scientific work on migration detention in the *estaciones migratorias* and its impacts is scarce, various non-governmental organisations have been reporting inhumane conditions and human rights violations of migrants in Mexican migration detention for a long period (Colectivo Contra la Tortura y la Impunidad, 2019; Consejo Ciudadano del Instituto Nacional de Migración, 2017; Insyde, 2017; Sin Fronteras, 2016). In some cases, they might even amount to torture (Comisión Mexicana de Defensa y Promoción de los Derechos Humanos, 2019). Valadez and Echenique (2016) and Sin Fronteras (2019) conceptualise *estaciones migratorias* as spaces of impunity where systematic ill-treatment stays without any juridical consequences. Pérez Bravo (2020) indicates a failure in medical and mental health services across all detention facilities. Fernández de la Reguera Ahedo (2020) articulates that the treatment of detainees and their experience of the *estaciones migratorias* differ according to their gender and nationality. In line with this, an exploratory study with former detainees indicates intersectional differences regarding the treatment and conditions between distinct detention centres (Manek, 2019).

### Mental health, migration detention and the configuration of detention environments

In general, refugee and migrant populations do have worse mental health outcomes than citizens (Abbas et al., 2018). Migrant detainees show even higher levels of PTSD, anxiety disorders and depression compared to the non-detained population that last even beyond the detention period (Cleveland et al., 2018; Steel et al., 2006). Especially children who had not shown psychological symptoms before detention develop new mental and physical health difficulties within detention (Lorek et al., 2009; Mares, 2016). Whether the length of detention is per se a detrimental factor to mental health remains under discussion. Yet, two systematic reviews assume a direct relationship (Robjant et al., 2009; von Werthern et al., 2018). In their systematic review on immigration detention in the UK, the US and Australia, Robjant et al. (2009) emphasised that the time in immigration detention contributed even to mental health difficulties when other significant risk factors such as prior trauma were taken into account. However, empirical evidence of the detrimental effects of detention in the *estaciones migratorias* is rare in clinical research and little is known about interdisciplinary interconnections. The aim of this research is to further explore this nexus and its underlying components—the environment of the detention facilities, the detainee’s mental health and the broader social and political context.

Based on the reports of human rights organisations, our main hypothesis is that the *estaciones migratorias* are torturing environments: The concept of the torturing environments (Pérez-Sales, 2016) puts in relation clusters of basic human needs (primary physiological functions, relation to the environment, need for safety, physical integrity, self, and identity) with different types of attacks inflicted and its consequences produced (affect and anxiety circuits, higher functions). The concept of torturing environments challenges the perception of torture as directed foremost against physical integrity. Instead, it might not leave physical marks but has a severe impact on the detainees’ mental health and social relations. Thus, we suppose that a connection exists between the particular configurations of the torturing environment and the reported mental health of the interviewees: We assume that the more severe a torturing environment and the longer the duration of detention, the worse the emotional constraints. The *estaciones migratorias* concentrate detainees in separated areas according to their gender differences. While von Werthern et al. (2018) stress that studies on immigration detainees mainly focus on cis-male samples, having a female gender might imply a higher risk for developing emotional constraints in detention environments (Young and Gordon, 2016). In general, female migrants face continued exposure to sexual maltreatment within host countries (Lay & Papadopoulos, 2009). Regarding migration detention in Mexico, the explorative study on *estaciones migratorias* (Manek, 2019) and reports of human rights organisations indicated intersectional differences in the treatment of detainees (Consejo Ciudadano del Instituto Nacional de Migración, 2017; Fernández de la Reguera Ahedo, 2020; Sin Fronteras, 2019). Therefore, we suppose that the configuration of a potential torturing environment might differ between male and female interviewees, especially regarding (5) *attacks against sexual integrity*. In addition, we assume that emotional constraints and might differ between the two groups. Immigration detention is supposed to be an acute stressful condition. In line with the results of Keller et al. (2003), we hypothesise that released participants show lower levels of mental health constraints than those who are still in detention. In addition, the two groups might report different environmental configurations. As said before, because existing research is still scare, all hypotheses are also explored qualitatively. Eventually, the configuration of *estaciones migratorias* as a potential torturing environment is looked at from an interdisciplinary perspective: are they systematically structured and how are they situated in terms of the broader system of Mexican migration detention politics?

## Methods

In order to investigate the impact of the Mexican *estaciones migratorias* on the detainees’ mental health and its nexus with the social relationships of migrant detainees, we developed an interdisciplinary and mixed method approach, organised in two steps.

**Fig. 1:**
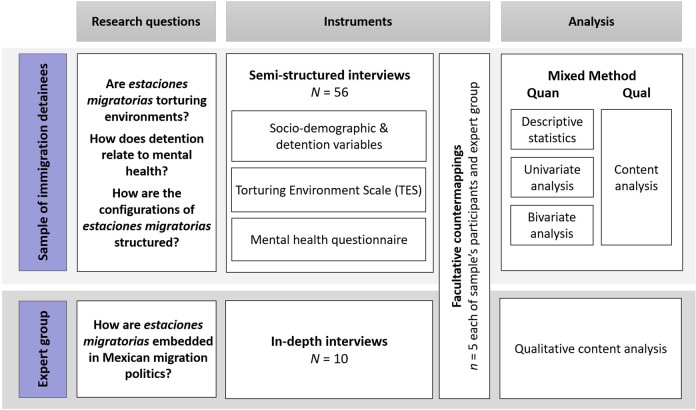
Research design. Representation of the mixed-method design, differentiating between the semi-standardised interviews with the sample of (former) migrant detainees and the in-depth interviews with the expert group. The figure portrays the respective research questions, instruments and methods of analysis. This figure is covered by the Creative Commons Attribution 4.0 International License. Reproduced with permission of Julia Manek; copyright © Julia Manek, all rights reserved.

In a first step, the mixed method core of this research explores the above-explained hypothesis and open questions, based on a cross-sectional study with a sample of immigration detainees (see Fig. 1). An embedded design (Creswell, 2007) combines both the quantitative and qualitative data which explores different aspects of the detention environment and mental health. The mentioned dataset is collected in semi-structured interviews which were organised along the dimensions of the TES. In a second step, we embedded the findings of the detainees’ sample into a broader scope: Based on in-depth interviews with an expert group of key actors in human rights and migration detention in Mexico, we investigated how migration detention affects social relationships on a bigger scale and how the configurations of detention centres are embedded in the broader landscape of migration politics.

### Sample

In total, *N* = 56 participants were interviewed who had either been released from an *estación migratoria* or were interviewed while still being detained. The majority of participants were interviewed after release while living in civil society shelters. In 22 cases, the interviews were conducted within detention. Most detentions took place between the exodus in 2018 and the outbreak of the pandemic in 2020. Eight interviewees had been detained in 2017.

In total, there is data on eleven different detention centres, with people mainly being detained in two centres: Tapachula and Mexico City. The interviewees were detained for an average of 51.6 days, varying from a minimum of one day to a maximum of 270 days. Most of the interviewees were adult men. Participants mainly hold citizenships of Honduras, followed by Cuba, El Salvador, Guatemala and five other nationalities as described in Table 1. The educational level varied from primary school to university formation. More than half of the main sample reported conditions of vulnerability, such as having minors in their care, belonging to the LGBTIQ population, experiencing language barriers or having serious health problems or a disability.

**Table 1:** Characteristics of the sample.

Detention status *n* (%)	Detained	22 (39.29%)
	Released	34 (60.71%)
Detention centre	Tapachula	22 (39.29%)
	Mexico-City	21 (37.50%)
	Other	13 (23.21%)
Days in detention mean (s.d.)		51.6 (58.8)
Sex *n* (%)	Men	44 (78.57%)
	Women	12 (21.43%)
Age mean (s.d.)		30.18 (9.63)
Nationality	Honduras	24 (42.86%)
	Cuba	12 (21.43%)
	El Salvador	9 (16.07%)
	Guatemala	4 (7.12%)
	Nicaragua	3 (5.36%)
	Pakistan	1 (1.79%)
	Nigeria	1 (1.79%)
	Columbia	1 (1.79%)
	Dominican Republic	1 (1.79%)
Level of education *n* (%)	Primary	14 (25.00%)
	Secondary	10 (17.86%)
	Tertiary	14 (25.00%)
	Unknown	18 (32.14%)
Vulnerability	Yes	24 (42.86%)
	No	11 (19.64%)
	Unknown	21 (37.50%)
Asylum application	Yes	45 (80.36%)
	No	5 (8.93%)
	Unknown	6 (10.71%)

Total *N* = 56.

### Instruments

To address the impact of the migration detention centres, this research builds mainly on the first section of the *Torturing Environment Scale* (TES, Pérez-Sales, 2016). The TES is a validated instrument that indicates whether an environment can be considered torturing. Its original version was published in Spanish. Translations into English and French are available.

The TES’ main section consists of an *Assessment of the Environment*. It bears eight subscales, which analyse the environment of the migration detention centres: (1) *contextual manipulations*, (2) *fear-producing actions*, (3) *pain-producing actions*, (4) *extreme pain*, (5) *sexual integrity*, (6) *need to belong*, (7) *actions targeting identity and sense of control* and (8) *interrogatory*. A validation study determined categorical omega values *ωc* as indicators for internal consistency ranging from 0.44 to 0.72 (Pérez-Sales et al., 2021). Based on the standard of reference in the assessment of torture allegations (which is the Istanbul Protocol, United Nations, 2004), expert assessments confirmed the TES’ convergent validity (Cakal, 2018; Jaranson, 2017), which suggests the robustness of the scale.

Taken together, 44 items of the eight above-mentioned subscales were included with either four or eight items each. Total values were calculated for each subscale. Values in single TES subscales of five or more indicate the existence of a torturing environment. Yet, it is important to notice, that the TES is not supposed to measure the suffering of persons (Pérez-Sales et al., 2021). Its indication of exposure to a torturing environment can be bound to a person having suffered from torture but does not necessarily equal it.^4^ Still, the TES allows to explore correlations with clinical variables.

In order to assess the potential torturing environments, the TES includes an evaluation of the detainees’ mental health (Pérez-Sales, 2016). For the particular environment of the Mexican *estaciones migratorias*, an expert group of clinical psychologists and human rights actors developed a questionnaire on mental health and emotional distress based on the psychometric structure of the Assessment Schedule of Serious Symptoms in Humanitarian Settings (WASSS, World Health Organization, 2012). According to their judgement, 13 items were included (Grupo Impulsor Contra la Detención Migratoria y la Tortura, 2020). All items are represented in Table 2: they indicate mental or somatic health constraints that were in line with validated instruments assessing PTSD, depression and anxiety disorder. A trained psychologist and a Spanish native speaker transcribed the resulting questionnaire into Spanish.

**Table 2:** Mental health criteria.

		Mean	s.d.
1	Fatigue *n* (%)	2.80	0.94
2	Sadness *n* (%)	2.66	1.1
3	Nightmares, intrusive thoughts or images *n* (%)	1.96	0.92
4	Mistrust *n* (%)	2.57	1.08
5	Anger or rage towards self or others *n* (%)	1.89	0.95
6	Guilt *n* (%)	1.91	1.04
7	Fear *n* (%)	2.57	1.09
8	Anguish and despair *n* (%)	2.63	1.14
9	Despair *n* (%)	2.32	1.22
10	Suicide (specify) *n* (%)	1.20	0.52
11	Humiliation or shame *n* (%)	2.20	0.98
12	Moments of happiness in spite of everything *n* (%)	1.84	0.76
13	Other stressors *n* (%)	2.46	1.22

Total *N* = 56.

### Statistical and qualitative analysis

**Fig. 2:**
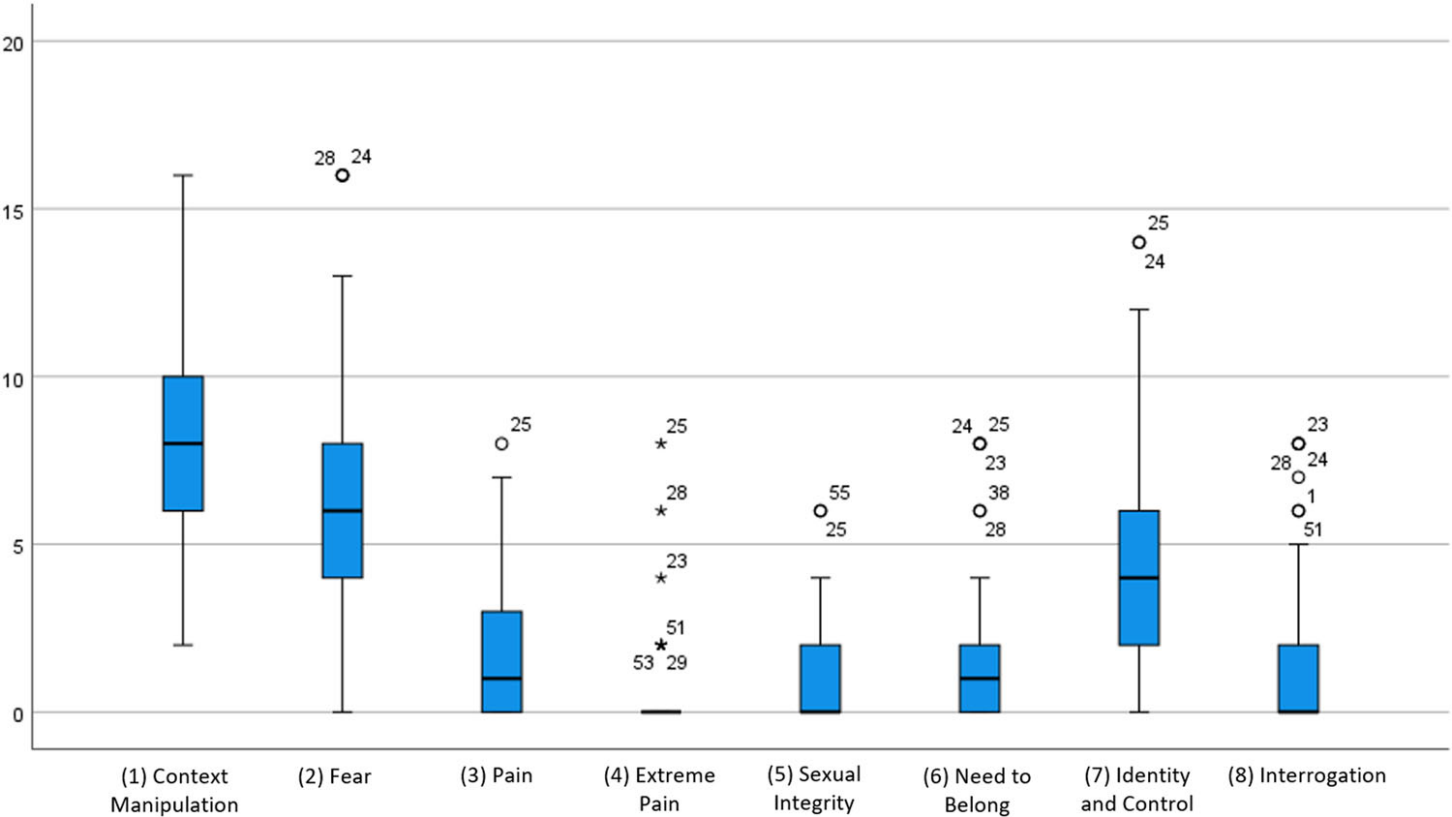
Median and IQR of the TES subscales. The figure displays boxplots of the eight subscales. Statistical extreme values are marked with asterisks. This figure is covered by the Creative Commons Attribution 4.0 International License. Reproduced with permission of Julia Manek; copyright © Julia Manek, all rights reserved.

Figure 2 shows descriptive statistics of the TES subscales, as well as frequencies of options and cut-off criteria indicating a torturing environment. For an overview of the psychological stressors and environmental factors, frequency counts of the individual items were prepared (see Tables 2 and 3).

**Table 3:** Overview of all items [subscales of the TES].

		Mean	s.d.	*n* ≥ 5
(1) Context manipulation		8.25	3.51	47
a	Inhuman conditions	1.70	0.60	
b	Manipulation of environmental conditions	1.61	0.76	
c	Altering basic physiological functions	1.41	0.8	
d	Sleep dysregulation	1.09	0.86	
e	Manipulation sense of time	0.87	0.88	
f	Partial deprivation of senses	0.21	0.59	
g	Medical induction of altered states	0.21	0.56	
h	Other contextual manipulations	1.14	0.96	
(2) Fear		6.27	3.86	36
a	Manipulation of hopes and expectations	1.46	0.76	
b	Threats against the person	1.18	0.9	
c	Threats against family	0.70	0.87	
d	Anguish associated with lack of information	1.16	0.87	
e	Experience of near death	0.20	0.55	
f	Forced witnessing of other’s torture or death	0.39	0.78	
g	Use of situations evoking insurmountable fear	0.25	0.61	
h	Other situations provoking fear or terror	0.93	0.99	
(3) Pain^a^		3.32	3.76	17
a	Blunt trauma	0.66	0.84	
b	Forced battles against oneself	0.68	0.9	
c	Exhaustive exercises	0.14	0.48	
d	Other pain-producing actions	0.18	0.54	
(4) Extreme pain^a^		1.00	2.98	3
a	Devices that produce excruciating pain	0.16	0.53	
b	Mutilation	0.11	0.41	
c	Brain damage	0.13	0.47	
d	Other actions producing extreme pain	0.11	0.41	
(5) Sexual integrity^a^		1.96	2.82	4
a	Humiliation related to sexual identity	0.20	0.55	
b	Sexual assault	0.16	0.5	
c	Rape	0	0	
d	Other actions targeting sexual integrity	0.63	0.82	
(6) Need to belong^a^		3.39	4.3	12
a	Prolonged solitary confinement	0.36	0.75	
b	Breaking social bonds	0.70	0.81	
c	Manipulation of affect	0.29	0.65	
d	Other actions targeting the need to belong	0.36	0.72	
(7) Identity and control		4.21	3.40	24
a	Attacks on sense of self	0.41	0.65	
b	Induced submission and compliance	1.13	0.83	
c	Instilling guilt	0.46	0.74	
d	Induced shame	0.75	0.84	
e	Induced humiliation	0.93	0.85	
f	Violation of taboos	0.27	0.56	
g	Installing goals and identity	0.14	0.44	
h	Other actions targeting identity	0.13	0.43	
(8) Coercive interrogation		2.82	4.70	11
a	Extreme conditions during interrogation	0.45	0.78	
b	Conditions of interrogation that foster false confessions: extreme emotions	0.38	0.70	
c	Conditions of interrogation that foster false confessions: lies or deliberate confessions	0.25	0.61	
d	Other extreme coercive actions	0.29	0.66	

Total *N* = 56. Values in the TES subscales of ≥5 indicate the existence of a torturing environment as defined by the TES.

To assess the relationships between emotional constraints and the environment of the *estaciones migratorias*, bivariate correlations were calculated. To explore possible differences in dimensions of the torturing environment, we tested group differences for the following independent variables: detention centres (Tapachula vs. Mexico-City vs. others), the status of detention (released vs. detained) and gender (male vs. female)^5^. Due to the violation of normal distribution for single subscale means, we performed bivariate analyses using the non-parametric Kruskal–Wallis tests for all factors (*k* ≥ 2 levels; Dalgaard, 2002). We included (a) the TES subscales, (b) the time in detention and (c) the mental health criteria. Standardised effect size estimates *r* was calculated for group differences in conceptual block scores. The level of significance was set at *p* < 0.05, and Bonferroni correction was used when analysing differences between categories of independent variables. All statistical analyses were carried out using SPSS v27.

The quantitative findings are prone to interpretation biases and a generalisation to underlying populations beyond this sample. Although this research counts with the official translation of the TES and a cautious translation of its emotional questionnaire, transcultural measurement issues should be considered as a potential source of error, especially in the two cases of people not speaking Spanish. Yet, the integration of quantitative and qualitative data as an embedded design is expected to buffer such biases, as all interviewees had the opportunity to further elaborate on their thoughts on the questions of the TES in the semi-structured interviews. A thematic analysis based on the dimensions of the TES processed the qualitative data.

All interviews were transcribed with help of the software f4transcript. The evaluation was executed with the help of the qualitative data analysis software MAXQDA (Kuckartz and Rädiker, 2019).

### Expert interviews

In order to integrate the findings of the sample on a greater scale within the Mexican migration detention landscape, additional in-depth interviews were conducted with an expert group regarding Mexican immigration detention. These last interviews complemented the semi-standardised interviews of migrant detainees. Ten key actors in migration detention and human rights were interviewed, either because they monitored *estaciones migratorias* or did interdisciplinary research on them. The purposive sampling strategy for the expert group aimed at maximising the variance: We included five human rights defenders who monitored particular *estaciones migratorias* that had not been part of the scope because none of the sample’s participants had been detained there. In-depth interviews with scholars of different disciplines (sociology, economic sciences, psychology, psychiatry, and legal studies) embedded the analysis of particular detention centres into a more systematic perspective of migration politics, torture and equality. All interviews unfolded from the dimensions of the TES as a starting point and developed according to the interviewees’ emerging perspectives. Transcription and analysis of the expert interviews were analogous to the interviews with participants of the sample.

### Counter-mappings

In part, interviews both with participants of the immigration detainees’ sample and the expert group were accompanied by a facultative psychogeographical counter-mapping of the detention space—a novel interdisciplinary research method derived from critical migration, feminist and psychologist research (Campos-Delgado, 2018; Manek and Fernández de la Reguera, 2022) to assess the environments of the *estaciones migratorias* spatially and visually. The interviewees were asked to draw a mental map (Gieseking, 2013; Kitchin, 1994) of their spatial memories of the *estación migratoria* and their particular experiences in the different areas as if they would draw a site plan: In which areas of the detention centre did potential attacks to the self or to basic physiological functioning occur and which feelings are connected to it? Physiological sensations, emotions and particular experiences were represented via different colour codes and icons. Taken together, this research builds on each *n* = 5 counter-mappings of both the sample of (former) detainees and the expert group.

### Data collection

The data collection with a sample of (former) immigration detainees took part between the arrivals of the first caravans from Central America in Mexico and the rise of the Covid-19-pandemic, from October 2018 to March 2021. Specially trained human rights defenders of the Grupo Impulsor Contra la Detención Migratoria y la Tortura, including the first author, conducted the interviews. The interviewers were schooled to be sensitive toward the asymmetrical relation between interviewer and interviewee, given the vulnerability created in the context of forced migration and especially detention (e.g. Maillet et al., 2017; Nungsari et al., 2020). Guidelines were established that allowed the interviewee to lead the interview as a person who has had experience of immigration (Grupo Impulsor Contra la Detención Migratoria y la Tortura, 2020). All interviewers attended a full training weekend on the dimensions of the TES, including trauma-sensitive psychological first aid interventions. The role of the interviewers consisted of monitoring the development of the interview by creating a respectful and trusting space, despite the fact that many of the interviews took place in immigration detention centres under constant surveillance by *INM* agents and other police and military forces. Interviewees were primarily selected according to their willingness to volunteer, but also regarding apparent emotional stability. If participants did not speak Spanish, they were interviewed in English instead. Interviews with (former) detainees were conducted by different trained interviewees belonging to the research team, including the first author. All participants of the sample received a contact of the research group and could request a follow-up session if the interview should have triggered reactions of emotional distress.

The interviews with the expert group took part in August and September 2018 and then again in May and June 2021, that is before and after the interviews with the sample. The first author conducted all expert interviews of the mixed sample as well as the counter-mappings.

## Findings

This section reports all findings. At first, mixed method results of the interviews with (former) detainees are displayed. We present the quantitative results, derived from the sample’s semi-structured interviews to then integrate them into the qualitative findings. Finally, we present the findings derived from the interviews with the expert group to embed the sample’s previous results on detention experiences into the broader framework of migration detention and migration politics in Mexico.

### Sample-based findings: *estaciones migratorias* as torturing environments?

The following findings describe torturing environments as measured by the TES. Figure 2 plots the means and standard deviations for the subscales of the TES. Table 3 shows the tendencies of the answers per item according to all subscales. Figure 2 and Table 3 show the mean scores and variance of all subscales.

The subscale (1) *context manipulation* has the highest mean score across all subscales. All interviewees stated that they had been exposed to some kind of manipulation of the detention environment. It was reported that the cells did not meet the minimum conditions: they were overcrowded, they had no place to sleep or lacked hygiene. Likewise, respondents stated that they were subjected to the manipulation of environmental conditions, such as extreme temperatures or humidity. Most of the interviewees reported an alteration of their basic physiological functioning based on external factors like hunger, thirst, being limited in their ability to defecate or suffering from sleep dysregulation.

Most of the 56 respondents reported (2) *fear* (92.9%) and manipulation of expectations and hopes. Anguish associated with the lack of information about family members in detention was referred to in particular. (3) *Pain* was referred to frequently (57.1%), among other things, people reported having been beaten. Another five people described sustaining injuries without medical attention and being attacked by agents with tear gas or pepper spray. Although very few people reported events of (4) *extreme pain*, they occurred within the Mexican migration detention: two persons reported mutilation or brain damage.

Nearly half of the respondents (48.2%) reported forms of humiliation related to their (5) *sexual integrity*. In the context of the (6) *need to belong*, acceptance and affection, and isolation from an effective environment were the most reported by almost half of the group. Eight persons were reported to have experienced a period of solitary confinement, one person of more than 15 days. Regarding the question of (7) *identity and control*, the majority of interviewees (83.9%) reported helplessness and obedience-inducing techniques, e.g. changing rules or the use of punishment. In addition, shame and humiliation were induced, e.g. by preventing personal hygiene or by insults. Although few events on (8) *coercive interrogation* became evident, the application of conditions which favoured false confessions, e.g. by provoking emotional exhaustion, emerged in interrogation situations.

Both Fig. 2 and Table 3 indicate that the environment of the *estaciones migratorias* consists of forms of a torturing environment, primordially based on a manipulation of the environmental conditions. The high number of cases that surpass the cut-off value of the TES subscales indicates exposure to a substantially torturing environment. It is eminent that the mental health of detainees in estaciones migratorias is heavily affected (see Table 2). It seems that the majority of the detainees feel a devastating impact independent of differences between the immigration detention centres, e.g. in terms of detention conditions or treatment. As the TES is “by no means a measure of the suffering of persons and should not be used as such” (Pérez-Sales et al., 2021, p. 9), the statistical results will be embedded in and explained along with the qualitative data in the following.

### Mental health

The interviews with the sample participants sketched a fierce relationship between mental health and detention. Most of the respondents reported feeling humiliated or ashamed (89.3%). Over three-quarters of the sample reported fear, as well as experiencing anguish and despair at least once a week (78.9%). In addition, the majority described experiencing fatigue at least three days a week (64.9%). Other outlined negative feelings such as frustration, worrying about the future, uncontrolled crying, lack of concentration and frequent sleep disturbances. In general, many mental health issues were reported across the section of mental health criteria. Table 2 shows the descriptive values of the mental health of the main sample.

### Interactions: correlations and group differences

This section reports interactions between the TES subscales, demographic characteristics, detention place and mental health criteria. With few exceptions, significant to highly significant correlations with medium to large effect sizes prevailed between the subscales of the TES. Table 4 shows bivariate correlations between the eight subscales of the TES, the time spent in detention and the mental health measures. Time of detention correlated significantly with (3) *pain*, (4) *extreme pain*, (6) *need to belong* and (8) *coercive interrogation*. With the exception of subscale (5) *sexual integrity*—for which there was surprisingly no effect—the scores of all other subscales of the TES correlated strongly with the reported mental health constraints. Surprisingly, we did not observe a correlation between mental health and the time spent in detention.

**Table 4:** Pearson correlation coefficients for TES subscales, time in detention, and mental health criteria.

Variable	1	2	3	4	5	6	7	8	9
1 Context manipulation									
2 Fear	0.51^**^ ^[0.21, 0.71]^								
3 Pain	0.57^**^ ^[0.31, 0.74]^	0.66^**^ ^[0.43, 0.79]^							
4 Extreme pain	0.48^**^ ^[0.15, 0.66]^	0.47^**^ ^[0.01, 0.69]^	0.66^**^ ^[0.23, 0.82]^						
5 Sexual integrity	0.21 ^[−0.09, 0.49]^	0.34^*^ ^[0.05, 0.59]^	0.29^*^ ^[−0.07, 0.59]^	0.39^**^ ^[−0.07, 0.72]^					
6 Need to belong	0.42^**^ ^[0.06, 0.66]^	0.49^**^ ^[0.15, 0.70]^	0.42^**^ ^[0.05, 0.66]^	0.59^**^ ^[0.05, 0.77]^	0.34^*^ ^[0.02, 0.63]^				
7 Identity and control	0.68^**^ ^[0.49, .80]^	0.73^**^ ^[0.53, 0.85]^	0.70^**^ ^[0.54, .82]^	0.54^**^ ^[0.15, 0.75]^	0.44^**^ ^[0.15, 0.66]^	0.59^**^ ^[0.23, 0.78]^			
8 Coercive Interrogation	0.65^**^ ^[0.41, 0.79]^	0.52^**^ ^[0.18, 0.73]^	0.63^**^ ^[0.33, 0.78]^	0.63^**^ ^[0.15, 0.82]^	0.21 ^[−0.11, 0.55]^	0.59^**^ ^[0.20, 0.79]^	0.64^**^ ^[0.37, 0.80]^		
9 Time in detention	0.18 ^[−0.15, 0.82]^	0.26 ^[−0.03, 0.50]^	0.33^*^ ^[0.07, 0.53]^	0.33^*^ ^[−0.02, 0.59]^	0.08 ^[−0.17, 0.38]^	0.53^**^ ^[0.28, 0.78]^	0.20 ^[−0.14, 0.50]^	0.40^**^ ^[0.08, 0.61]^	
10 Mental health criteria	0.71^**^ ^[0.53, 0.82]^	0.43^**^ ^[0.12, 0.64]^	0.46^**^ ^[0.22, 0.64]^	0.44^**^ ^[0.14, 0.61]^	0.08 ^[−0.29, 0.39]^	0.47^**^ ^[0.20, 0.66]^	0.59^**^ ^[0.31, 0.73]^	0.43^**^ ^[0.14, 0.63]^	0.05 ^[−0.27, 0.35]^

*N* = 56.

Values in square brackets indicate the 95% confidence interval for each correlation.

*p < 0.05, **p < 0.01.

As expected, different detention centres seem to vary in (1) *the conditions* of the detention context. This is also the case regarding attacks on (7) *identity and self-control*. Substantial differences always and exclusively manifested between the detention facility of Mexico-City and the cluster of different centres (see Table 5).

**Table 5:** By detention centre.

Median (IQR)	Tapachula (*n* = 22)	CDMX (*n* = 21)	Other (*n* = 13)	Test statistic	Effect size estimate (*r*_a_)
(1) Context manipulation	8 (7–10)	6 (4–9)^a^	9 (8–16)^a^	7.986*	−2.70
(2) Fear	6 (2–8.25)	8 (4.5–8)	7 (4.5–13)	1.647	–
(3) Pain	4 (0–6)	0 (0–4)	4 (0–8)	3.052	–
(4) Extreme pain	0 (0–0)	0 (0–0)	0 (0–0)	5.042	–
(5) Sexual integrity	0 (0–2)	2 (0–4)	4 (0–4)	4.683	–
(6) Need to belong	2 (0–4)	2 (0–4)	4 (0–14)	4.393	–
(7) Identity and control	4.5 (2–6.25)	2 (1–4)^a^	6 (3–11)^a^	8.178*	0.02
(8) Coercive interrogation	0 (0–2.5)	0 (0–3)	2 (0–15)	3.230	–
Time in detention	16.50 (10–63.25)	29 (22–46)	30 (13.5–150)	2.015	–
Mental health criteria	31 (28–33.5)	28 (20–36)	35 (23–46.5)	3.154	–

^a^The superscript letter marks those groups between which significant differences manifest.

*p-values < 0.05.

No substantial differences manifested between male and female detainees on the TES subscales or on mental health constraints, as reported in Table 6.

**Table 6:** By gender.

Median (IQR)	Male (*n* = 45)	Female (*n* = 12)	Test statistic	Effect size estimate (*r*)
(1) Context manipulation	8 (5–10)	9.5 (7.25–11.75)	3.027	–
(2) Fear	6 (3.5–8)	7 (5.25–8.75)	1.898	–
(3) Pain	4 (0–6)	0 (0–4)	1.037	–
(4) Extreme pain	0 (0–0)	0 (0–3)	1.769	–
(5) Sexual integrity	0 (0–4)	11 (0–4)	0.117	–
(6) Need to belong	2 (0–4)	3 (0–7)	0.201	–
(7) Identity and control	4 (1.5–6)	4,5 (1.25–7.75)	0.284	–
(8) Coercive interrogation	0 (0–3)	1 (0–7)	0.966	–
Time in detention	26 (12.5–101)	21 (10.75–29.95)	0.848	–
Mental health criteria	29 (20.5–35)	35 (28.25–38.5)	3.884	–

Total *N* = 56

In comparison to interviewees who had already been released, considerably more attacks on the (7) *self and on identity* were reported by interviewees who were still in detention (see Table 7). Analogous to this, interviewees who had been released from detention and spoke in shelters about their detention experience were expected to report significantly less emotional constraints than interviewees who were interviewed during detention. However, no significant differences were found.

**Table 7:** By status of detention.

Median (IQR)	Released (*n* = 34)	In detention (*n* = 22)	Test statistic	Effect size estimate (*r*)
(1) Context manipulation	8.5 (4.75–10)	8 (7–12)	1.662	–
(2) Fear	6 (3.75–8)	6.5 (4–9.25)	2.280	–
(3) Pain	1 (0–6)	4 (0–6)	0.698	–
(4) Extreme pain	0 (0–0)	0 (0–1)	2.431	–
(5) Sexual integrity	0 (0–2.5)	1 (0–4)	0.534	–
(6) Need to belong	2 (0–4)	2 (0–9)	1.165	–
(7) Identity and control	3 (1–5.25)	5 (2.75–8)	6.392**	0.85
(8) Coercive interrogation	0 (0–2.5)	2 (0–5)	2.657	–
Time in detention	24 (14.5–35.75)	30.5 (10–150)	0.636	–
Mental health criteria	29.5 (20–36)	32 (27.5–36.25)	1.306	–

Total *N* = 56.

**p < 0.01.

### Qualitative integration: configurations of *estaciones migratorias* as torturing environments

**Fig. 3:**
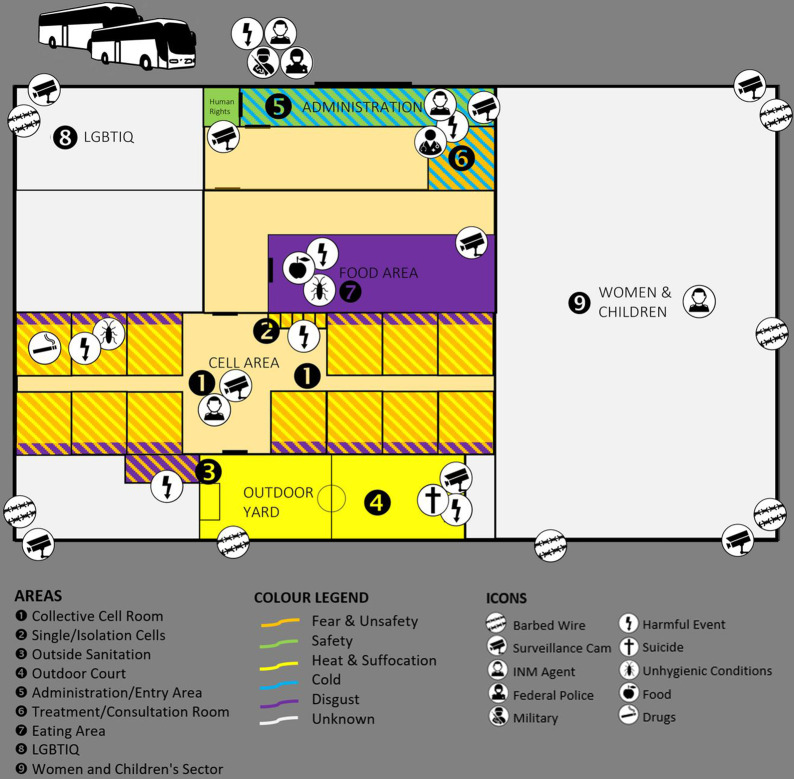
A spatial configuration of a torturing environment. This figure is a visual integration of the different counter-mappings by *n* = 5 former detainees and *n* = 5 participants of the group of key actors. The integration portrays an estación migratorias from the perspective of an adult cis-male inmate. The figure indicates different areas. Colours represent emotions linked to a certain place. Icons describe the infrastructure, conditions of the detention’s environment, incidents and different state actors. This figure is covered by the Creative Commons Attribution 4.0 International License. Reproduced with permission of Julia Manek; copyright © Julia Manek, all rights reserved.

The qualitative findings meet with the descriptive results: a focus on (1) *context manipulation* is most important to understand the mechanisms of detention. Tables 3 and 8 report the highest mean score for this TES subscale. In this section, we will describe it in detail. Being the integrated result of the single counter-mappings, Fig. 3 reflects the manipulation of the detention environment visually. It illustrates the spatial configurations of the torturing environment across the different areas of the detention centre. The sample’s narrations of particular *estaciones migratorias* supported the statistical assumption of gradual differences between centres (Mexico-City vs. other centres). At the same time, similar configurations between the detention centres became evident in the qualitative data, which support the absence of statistical differences between the two main *estaciones migratorias* (Tapachula vs. Mexico-City) regarding the TES subscales: throughout nearly all of the interviews, it became clear that (1) *the manipulation of the context* and environmental conditions essentially produced inhuman conditions. The emerging landscape of dehumanisation is built on unhygienic conditions and sleep dysregulation. Figure 3 shows that they occurred especially in the cell areas and sanitary installations:

“There were only concrete ‘beds’, with up to three people on top of them, most of them slept on the floor. The place is small and we were about thirty people who got to be there. […] there was a lack of water in the toilets and this gave an atrocious smell.”

**Table 8:** TES subscale values and selected themes.

	No. of items	Min.	Max.	Mean	s.d.	*n* ≥ 5	Selected themes [quotations of sample’s participants]
(1) Context manipulation	8	2	16	8.25	3.51	47	“Everything I saw inside this detention centre was terrible because there were so many people, there was hardly enough food.”
(2) Fear	8	0	16	6.27	3.86	36	“I did not get any information about my legal process until 15 days prior to my release from the immigration station, that is after four and a half months of immigration custody and I didn’t know what was going to happen with me.”
(3) Pain	4	0	16	3.32	3.76	17	“I was in a forced position before entering the cell. For 6 h on my feet, with the threat of beatings if you couldn’t stand it. The people who could no longer stand it were beaten by the INM agents”.
(4) Extreme pain	4	0	16	1.00	2.98	3	“[In the moment of arresting] they grabbed me from above by the hair like an animal and insulted me and threw me down. I hit my face and my teeth, my fangs, […] so it bothers me to eat and I don’t eat for days because they are broken.”
(5) Sexual integrity	4	0	12	1.96	2.82	4	“At the time of arrest there is a moment of sexual harassment. The officers tell us that if we have sex they won’t arrest us.”
(6) Need to belong	4	0	16	3.39	4.30	12	“During many weeks, I could not communicate with anyone.”
(7) Identity and control	8	0	14	4.21	3.40	24	“It is impacting emotionally. It’s better to keep away from trouble, you’re kind of humiliated like that. A constant humiliation that you feel inside. You can’t even open your mouth, not even to ask for soap, not even to ask for water.”
(8) Interrogation	4	0	16	2.82	4.7	11	“If was threaten with forced disappearance [in the asylum interview].”

Total *N* = 56.

The interviewees reported the distribution of rotten food as a further alteration of basic physiological functions. Another manipulation of the environment consisted of the manipulation of the sense of time: in some *estaciones migratorias*, detainees were kept in cells without windows or with permanent artificial light, which created disorientation resulting in a feeling of losing track of time.

Beyond this, disorientations were manifold. A general denial of information produced an environment of (2) *fear*, culminating in feelings of complete helplessness. Figure 3 indicates that especially within the cells and inside the corridors, feelings of unsafety and fear prevailed. Surprisingly, the quantitative results had not shown a linear relationship between the time spent in detention and mental constraints. However, the narrative scenarios reflect the significant correlation with length of detention and other subscales, especially (6) *the need to belong*. They emphasise the central role of temporality, which appeared to be interspersed with the harmful imperative of prolonged waiting: detainees must wait every day—both for access to the scarce services and to get out of detention. The lack of information interacts with fear for their own future and the whereabouts of family members. Beyond that, it attacks (6) *the need to belong*:

“It looked like they were beating women and many men had wives and daughters on the other side, and many men were asking for information about what was going on.”

(2) *Fear* also interacts with (3) *pain*, as threats are directed against the physical integrity:

“Because I have these tattoos, they wanted to remove them themselves in immigration. They wanted to burn them because they said that I was a gangster.”

The production of fear is stronger, as the threats become real. Especially male detainees are forcibly witnesses of violence, e.g. in punishment against protests:

“We were shouting: ‘Please, they are human beings, don’t treat them like that, please.’ […]. They took them like that, […] grabbed by the hands, by the feet, imprisoned, naked, with their faces disfigured, all beaten. […] But they threw them to the ground and began to hit them with everything, with their fists and electrocuted them.”

Although (3) *pain* does not seem to be central in the narratives of the interviews, many people did not only report threats but having been beaten themselves. This included punching as well as being in forced positions for a prolonged period, especially during and after detention or deportation (see Fig. 3). (4) *Extreme pain* seems to be bound to a specific event, often at the moment of arrest or deportation or as a punishment for the resistance of detainees as the mentioned example above shows.

The quantitative findings did not support the assumption of gender-based differences regarding the experience of detention. Nevertheless, the qualitative statements depict the detention centre to be a highly gendered place that appears to produce different emotional constraints. Especially women (see Table 6) reported acts directed against their (5) *sexual integrity*:

“At the time of arrest there is a moment of sexual harassment. The officers tell us that if we have sex, they won’t arrest us”.

Such acts did also address people belonging to the LGBTQ community and targeted their (7) *identity and sense of control*:

“They took away his retroviral drugs and said ‘that’s what you get for not using a condom and for messing with men’. They shouted that he was HIV positive.”

Attacks on the self like this were accompanied by insults and mockery, but also via e.g. arbitrary changing of rules or the use of punishment as well as submissive obedience-inducing techniques. In addition, feelings of shame emerged across the detention population due to preventing personal hygiene. In general, the landscape of dehumanisation and criminalisation provoked feelings of guilt:

“Talking to my lawyers, or asking for support from Human Rights, made me feel guilty. Because of these attitudes, I was going to stay in detention longer and without seeing my son.”

Attacks against (6) *the need to belong* became not only most visible when thinking of solitary confinement, but also when focusing on the separation of families and the lack of information and communication with relatives or lawyers outside of the detention centre. This enforced disruption consisted also of actions targeting (7) *the sense of control*. Statistically, released interviewees reported significantly fewer attacks against (7) *the sense of control*. Yet, the qualitative findings suggest that their effects did not necessarily end with detention, but extended beyond detention. This is shown by the following example:

“We got on the bus, it was with a lot of sadness that I got on the bus [to be deported] because I didn’t see my husband, the children were crying loudly and saying that they needed their dad.”

Concerning (8) *interrogation* techniques the case of migration detention is special—as no interviewee reported interrogation techniques e.g. aiming at forced confessions. Yet, instead of forcing false confessions, interview situations between the detainees and the INM were created that would prevent people from claiming asylum.

### Expert interviews: *estaciones migratorias* as part of deterrence politics

This section connects the above-described findings of the detainees’ sample with the expert interviews: It aims at broadening the scope of the previous subjective findings that are mainly based on subjective experiences toward a more systematic scope of the configuration of *estaciones migratorias* as torturing environments. How are these environments spatially configured? How are single detention centres embedded in the broader scale of national migration politics?

Although the particular *estaciones migratorias* are part of a whole system of detention, partial distinctions exist—as the above-presented mixed method findings indicate. Substantial differences have been shown concerning (1) *the contextual manipulation* and (6) *the need to belong*. As said before, statistical differences had emerged between detention centres regarding some subscales of the TES. And migrant detainees described differences in the configuration of torturing environments across different *estaciones migratorias*. Although each interview with monitoring experts focused on a different *estación migratoria*, all interviews stressed the multiple injuries that detention in *estaciones migratorias* inflicts on the detained population. Their interviews gave a clue as to why neither a correlation between the detention time and the emotional constraints of the detainees emerged nor differences between the two groups of detainees (released vs. detained) regarding their mental health: dehumanisation starts right at the gate. Already the arrest and the first hours in an *estación migratoria* may represent a profoundly and lasting shattering experience (see Fig. 3).

Human rights actors emphasised the re-traumatisation and recurring suicidal thoughts detention causes. Inside detention, suicides themselves are not rare. Often, the detainees are exposed to the dead bodies of those who committed suicide, as captured in Fig. 3. A human rights defender shared his monitoring experience from an *estación migratoria*:

“There is a burnt dungeon where a migrant who committed suicide and burnt some mats and they left him burnt [and others seeing his body], which is also a form of torture.”

Under the given condition, social spaces emerge in which the exposition of the suffering of others might affect the detainees further and hinder reciprocal relations:

“There was self-harm in one of the women. […] something that we can’t corroborate, is that she had like a psychiatric illness, I don’t know if that’s the right word, but she needed treatment and she wasn’t getting it. So it was like everything had gotten complicated and she was scratching her face, her arms.”

The different expert interviews pointed at intersecting factors leading to a veritable proliferation of illnesses. While some environmental manipulations are clearly intentional, others appear to be incidental—like the emergence of sickness. Yet, in the environments of *estaciones migratorias*, mental and physiological diseases arise on a structural level, e.g. through pathogenic food, humidity and extreme temperatures (Fig. 3 marks these structures mainly in purple and orange). In overcrowded conditions, infections spread between human bodies. Also, animals transmitted diseases: “There was no proper ventilation, so something that happened a lot, that was encouraged by the heat and that situation, was like this whole mosquito thing and so there were a lot of people who got sick with dengue fever.”

In addition, expert interviewees describe a systematic lacking or even harmful medical and psychological treatment. Different interviews indicated that the distribution of the already scarce resources was moderated by racism, worsening the exposition to harmful conditions:

“From Central American countries they saw that there was a little more privilege for Cuban people. […] For example, if they have illnesses, they don’t take the men out for medical attention and there is only one nurse […] who doesn’t manage to provide adequate care. There is no psychologist inside the migrant detention centre who can provide some therapy with this deprivation of liberty. So we have also noticed that there are many people who require psychiatric care and who are without medication, that is, who already have these problems from their country of origin and who are aggravated by being locked up for these prolonged periods of time.”

While different forms of individual punishment had emerged as topics in the sample’s interviews, the expert group indicated that the surrounding was also being used as a systematic punishment of the detained population as a whole:

“In the courtyard, there was a big metal door, which could cover, if you pulled it as if you were going to divide the space, it could cover the light of all the cells and shut down all the entrances at the same time. So, the people who were detained there were telling us that the station authorities used that giant metal door to punish them when there were riots or whatever and not to let them out.”

This culminated in the description of a particular detention facility. It seemed like the whole spatial structure is constructed to expose detainees to re-traumatising conditions:

“One of the most serious things that we noticed […] is that the facilities of the immigration station are adjacent to the facilities of the Federal Police. Not only are they like buildings next to each other, but this courtyard that I’m talking about has a fence that directly adjoins the shooting range of the Federal Police. […] People are listening all day long to the shots that are fired on the other side and at that time it was a very sensitive issue, because there were a lot of Cameroonian nationals who had just come out of conflicts, torture by police officers in their country and they were very stressed to be listening to all these shots. […] I think the psycho-emotional damage is very evident because of the conditions of listening to the detonations. It is terrible.”

All participants of the expert group stated unanimously that migration detention is a form of criminalisation and that the detention structure resembles imprisonment. While the torture committed by the staff and enforced by the environment’s manipulation were emphasised, a new dimension was reported, connected to the dehumanisation and criminalisation, that extended into the economical realm:

“Criminalisation is a business. In the case of Mexico, it is not because the estaciones migratorias are delegated to the private sector. […] It has an economic function because the more, in inverted commas, ‘illegal’ you are, undocumented you are, the less you are going to be able to demand your rights.”

As this last quote suggests, detention does not happen in a vacuum. Likewise, being part of a broader system, detention also represents a link in the chain of hurtful events of deterrence: arrest, detention and deportation (see Fig. 3 and Table 3). All were described as bound to physical violence and psychological threats that leave visible and invisible traces alike and beyond detention (see Tables 2 and 7). Detention and deportation attack belonging and separate families: This creates violent surroundings and aims at ending a migration journey—which tackles inequalities and claims rights. The subgroup of migration scholars foremost linked the creation of torturing environments in Mexican immigration detention to migration politics of deterrence that is displayed on the global scale.

## Conclusion

This work underlines that the Mexican migration detention system of the *estaciones migratorias* creates torturing environments and thus subsequently leads to multiple injuries. The results of this interdisciplinary and mixed method study emphasise that (1) *context manipulation* occurs in the *estaciones migratorias* that intersect with abusive treatment, especially creating (2) *fear* and attacks on (6) *the need to belong*. Yet, gradually different configurations emerged between particular centres. Statistically, no differences manifested between male and female interviewees regarding the experience of the detention environment and its mental health consequences. In contrast, the qualitative findings emphasise the gendered configurations of the institution. This contradiction between quantitative and qualitative results underlines the need to further strengthen an intersectional sensitivity if we want to accurately analyse torturing environments. Regarding the effects on detention across time, this study did not confirm the assumption of a linear relationship between the length of detention and mental health constraints. The absence of such a correlation did also prevail in other geographical contexts (e.g. Mares, 2016; Penovic, 2008), with Mares (2016) assuming a statistical ceiling effect. Moreover, the expected mental health improvement for released participants did not manifest. However, all released interviewees remained with the immigration status of registered asylum seekers—a precarious condition without temporary or permanent protection (Asylum Access, 2021). Momartin et al. (2006) and Coffey et al. (2010) show that the lack of legal protection hinders significant mental health improvement even after release from migration detention. Due to this, released detainees might have maintained an equal level of mental health constraints compared to detained participants. Given the persistently high level of mental stress and the worsening of attacks on (6) *the need to belong,* the longer the detention time, we must ask what other forms of harm detention causes—apart from mental constraints. Yet, the qualitative findings answer this question to a certain extent: they empirically underline the disastrous effects of perpetuated waiting and the negation of rights by various official practices to subjugate the migrants being detained. Such mechanisms have already been portrayed in other geographical contexts (e.g. Bhatia and Bruce-Jones, 2021; Isaacs, 2016; Mountz, 2011). Our findings indicate that detention is part of harmful deterrence politics which perpetuate inequality via the means of the detention environment and via the creation of feelings of fear and helplessness caused by detention and deportation. Although research must acknowledge that respondents may have exaggerated their reports, the coherence of the quantitative and qualitative findings suggests that accounts may have been accurate.

Future research should accompany the application of the TES with validated and translated clinical research instruments. The high differences in the means of the subscales of the TES with simultaneous high correlations between these subscales suggest that further research on the Mexican *estaciones migratorias* based on the TES should be refined at the item level. Aiming at an independent random sample with a broader number of participants, these instruments should sharpen the interpretation of results and allow a nuanced analysing of e.g. the differences between the environments of particular detention centres. In line with this, the qualitative approach pointing at intersectional differences should be integrated into quantitative designs.

The results of the expert interviews emphasise that detention does not exist in a socio-political vacuum. Its consequences extend into the social sphere and even into the economic sphere. In general, the breach between the measurements of the detention environment, its connections with mental health, as well as its social and political embeddedness requires a step-by-step elaboration of interdisciplinary and mixed method approaches. These interconnections need to be investigated further and ought to be researched across transnational migration regimes.

## Discussion

This study supports findings which show that detention enforcement seems not only to have a strong impact on mental health but that it also extends its impact on migration and the lives of migrants to broader spheres of societies. Former detainees continue the emotional aftermaths of detention. Even citizens who have never been detained themselves but personally know a formerly detained migrant is likely to report mental health constraints (Pinedo and Valdez, 2020). Clinical studies on migration often focus on traumatic experiences occurring in the context of pre-migration. Yet, post-migration factors such as detention may have an even stronger association with psychological distress (Carswell et al., 2011; Coffey et al.,^ 2010^; Schweitzer et al., 2006; Silove et al., 1997, 2000; Teodorescu et al.,^ 2012^). They should be analysed more deeply—in the context of deterrence politics and of the scale of whole societies.

Especially the intentional infliction of post-migration stressors such as the emergence of torturing environments should be discussed: although human rights organisations have documented systematic torture in migration detention all over the globe (e.g. Amnesty International 2019, 2020a, 2020b; Global Detention Project, 2014; Kiama and Likule, 2013), scientific research of torture and its implications for the detainees within migration detention is sparse and focuses mainly on the detention of torture survivors (Storm & Engberg, 2013). Only some studies show that detention conditions might have a torturing impact (Hárdi et al., 2016; Perez-Sales, 2016).

Ultimately, scholars analyse the US–Mexican border regime not only within the frame of deterrence politics but also of necropolitics (Inda, 2020; Trevino-Rangel, 2021): Following Mbembe (2008, 2016), necropolitical rule perceives racialised bodies as a form of property with a certain value. Those bodies can be kept alive but remain in a “state of injury”. Migration detention evolves as a continuity of postcolonial camps that destroys hope as much as human bodies (Mbembe, 2016). It immobilises migrants in a necropolitical limbo that makes it impossible to overcome existing inequalities, but keeps them in the realm of death (Butler, 2010; Dehm, 2020). In detention, migrants are initially created as fictionalised, detainable enemies, not only physically detained, but in a second step also dehumanised in the material reality of detention. Torturing environments intersect with the necropolitical ruling: According to Carney (2013), the social injury caused by migration detention centres goes hand in hand with the danger of migration detention’s social death and its long-lasting affective effects. Such effects consist of the production of fear, e.g. via the production of detainability and deportability (Conlon et al., 2017; De Genova, 2002, 2019)—and send a deterrent “message of non-welcome” which is projected to future migrations. Adaptivity is being normalised as a desirable behaviour—detainees long to leave detention, no matter in which (labour) conditions (Martin, 2021; Mezzadra and Neilson, 2013). The high probability to have lived through migration detention (Loyd et al., 2012), makes detention a crucial additional factor that leads to perpetuated precarity in the country of reception, in combination with general poor mental and physical health (Davidson and Carr, 2010).

Envisioning politics of deterrence on a global scale, Mountz (2020) outlines a triple “death of asylum” that includes not only the deaths of asylum seekers but also the ontological death of the idea of asylum and its death as a political-juridical category. In order to address these necropolitical landscapes and the inequalities resulting from them, scholars have made recommendations for interventions across all disciplines. However, in most cases, national states implement opposite measures which prevent access to safe and dignified living conditions even more and create dehumanising environments instead (Steel and Silove, 2004).

To counter this scenario, the concept of the *torturing environment* brings interdisciplinary perspectives together. It interweaves systematic knowledge of individualised subjects and particular places and allows to trace the material construction of a space of perpetuated injury. While torture addresses individuals, the torturing environment aims at populations. What does this knowledge of detention, dehumanisation and torturing environments—derived from local places—tell us about the construction of necropolitical spaces of deterrence within a global political theory? And, more importantly, how to overcome it?

## Data Availability

Due to the nature of this research, participants of this study did not agree for their data to be shared publicly, so supporting data is not available.

## Footnotes

1 The notion of the term “regime“, as it is used in this article, builds on the work of migration scholars that emphasises the transnational transformation and rescaling of states and societies: governance is not thought as a mere “top-down” process. The state may remain the main stakeholder in the emerging border regimes, but a multiplicity of other actors on multi-scalar levels play crucial and e.g. unpredictable roles in central political decisions, e.g. about whom to admit into national territory (e.g. Hess et al., 2018; Mezzadra and Neilson, 2013).

2 English translation: “migration stations”.

3 For example, in a temporal facility in Monterrey, the Subcommittee on Prevention of Torture noted dark rooms with no access to natural light or fresh air as well as spatial restrictions due to very small cells (Sin Fronteras, 2019). Insyde (2017) reported overpopulation, stale food and lacking or inadequate medical treatment in Vera Cruz. Detention could be extended indefinitely (Bahena Juárez, 2019).

4 Due to the length restriction of this paper, Section II (Intentionality), Section III (Legal As-pects) and Section (IV) Medico-Psychological Findings that allow to determine the exposition to torture are not reported in this study.

5 We controlled normal distribution via the Shapiro–Wilk test (Mohd Razali and Yap, 2011).
